# Supplementary material for: Utilisation of far infrared-emitting garments for optimising performance and recovery in sport: Real potential or new fad? A systematic review
Source: PLoS One. 2021 May 6;16(5):e0251282. doi: 10.1371/journal.pone.0251282 (PMC8101933; doi:10.1371/journal.pone.0251282)
Supplement: Attachment — Submitted filename: Responses to Editor and Reviewers-R1.docx [file pone.0251282.s002.docx]

Dear Editor of PlosOne,

Thank you for giving us the opportunity to revise our manuscript and to the reviewers’ comments. Accordingly, we have corrected the age range of participants included in studies selected in our systematic review and amended the discussion section to make it more succinct. Below is a point-by-point response to reviewers. We hope the article is now suitable for publication in PlosOne.

Yours sincerely,

Julien Louis

Reviewer #1: The present is a systematic review on far infrared-emitting garments for optimizing exercise performance. The review is clearly written and very interesting. I checked data in the literature and find that the review is complete. I also agree with the conclusion that more data are definitely needed on the effects of far infrared-emitting garments on exercise. Most importantly, the type of exercise must be standardized. I have only 2 comments:
a) the discussion is by far too long and can be extensively shortened.
b) at page 7, the authors state that age ranged between 18 and 35 years, but, being one of the subjects who tested this garment, I can tell you that unfortunately the age of participants is in some cases higher.

Many thanks for your review and relevant comments.

a) We have attempted to shorten the discussion, hence making it more succinct and strength to the point. All main paragraphs of the discussion have been amended accordingly, by removing sentences that were either repetitions or too hypothetical (with no sufficient scientific backup). These sections can be found in red in the revised manuscript and correspond to p12 L16-18; p12 L24-26; p13 L23-26; p15 L20-21 in the original manuscript. As a result, the discussion has been reduced by half a page.

b) thank you for pointing out this, it has been corrected with the full age range (18-57yrs) of participants in selected studies.

Reviewer #2: The effects of Far Infrared (FIR) emitting garments are not widely studied in the scientific literature to date. Thus, this review is of major interest in summarizing the effects of FIR emitting garments during exercise and recovery period. This review is well written, the methodology used is consistent and well informed despite the fact that few studies could be included in the review (n = 11). In their conclusion, the authors clearly highlight that FIR emitting garments could have an interest during exercise and recovery phase, however, additional studies must be carried out in particular with more subjects or more varieties of protocols.

Many thanks for your positive comments.
